# New Appliances and Things Medical

**Published:** 1903-12-19

**Authors:** 


### NEW APPLIANCES AND THINGS MEDICAL.

[We shall be glad to receive, at our Office, 28 & 29 Southampton Street Strand, London, W.C., from the manufacturers, specimens of all new preparations and appliances which may be brought out from time to time.]

### DAIRY REFRIGERATING MACHINES.

(H. J. West and Co., Ltd., Stamford Works, Southwark Bridge Road, London, S.E.)

Messrs. West and Co. have for many years been one of the foremost pioneers of modern refrigerating machinery and were one of the first to apply themselves to the refrigeration and cold storage of milk. By the rapid chilling of fresh milk, its susceptibility to atmospheric and other influences is vastly lessened, so that wide adoption of such a process should lead to material improvement in the public health. Both carbonic anhydride and ammonia compression machines are used, and as they take up little space, are easy to handle, and involve expenditure which would soon be recouped by the dairy owner by saving the waste which takes place under present circumstances; their use should certainly be more widely extended.

### WALL'S FOUNTAIN PEN.

Medical men and others in the habit of using a fountain pen, will find Wall's patent a useful and reliable variety. We find that it does not leak in the pocket if properly secured, and it becomes less clogged after a long period of rest than most pens of the kind. It is of good appearance and well finished.

